# Expressed emotion of caregivers among schizophrenic patients visiting Jimma University Medical Center Psychiatry Outpatient Unit, Southwest Ethiopia

**DOI:** 10.1186/s12991-022-00404-3

**Published:** 2022-07-18

**Authors:** Bethlehem Yimam, Matiwos Soboka, Yemiamrew Getachew, Bezaye Alemu, Gutema Ahmed, Elias Tesfaye, Mogesie Necho

**Affiliations:** 1 Department of Psychiatry, College of Medicine and Health Science, Wollo University, Dessie, Ethiopia; 2 Department of Psychiatry, College of Medicine and Health Science, Jimma University, Jimma, Ethiopia

**Keywords:** Ethiopia, Expressed emotion, Care givers, Schizophrenia

## Abstract

**Background:**

Expressed emotion (EE) measures the emotion among caregivers of schizophrenic patients and is predictive of symptom levels in a range of medical and psychiatric conditions. It is worth assessing expressed emotion and associated factors among caregivers of patients with schizophrenia in Ethiopia since there is limited data on this issue in this part of the world.

**Objective:**

To assess the status of expressed emotions and selected patients’ clinical factors among caregivers of patients with schizophrenia attending Psychiatry Outpatient Unit of Jimma University Medical Center, Southwest Ethiopia, 2019.

**Method:**

A cross-sectional study was conducted on 422 caregivers of schizophrenic patients using a consecutive sampling technique. Data were collected using a structured interviewer administrated questionnaires (Family Questionnairre) which assessed the level of expressed emotion. Data were entered into Epidata 4.4 and analyzed by Statistical package for social science (SPSS) version 25. Descriptive statistics was which used to summarize data, bivariate logistic regression was done to identify candidate variables for multivariable logistic regressions and the association between expressed emotion and predictor variables was identified by using multiple logistic regression models.

**Results:**

High expressed emotion was observed in 43.6% of respondents while caring for schizophrenic patients for about 6–8 years, having 3–4 episodes of the illness which was significantly associated with high expressed emotion.

**Conclusions:**

This study revealed that there is a high level of expressed emotion among caregivers. It also showed that increment in the episode of the illnesses had a significant association with high caregivers’ expressed emotion. Hence, health care systems which provide interventions for patients with schizophrenia need to design proper strategies to address caregivers’ needs as well.

## Introduction

Expressed emotion measures the emotion of caregivers and is predictive of symptom levels in a range of medical and psychiatric conditions [1]. Expressed emotion (EE) is an attitude, feeling, or behavior of the family caregiver in response to and reaction towards the person with schizophrenia [2].

The classification of expressed emotion in caregivers is based mainly on two variables; (1) ‘criticism’ (critical comments), and (2) emotional over-involvement, a third variable, ‘hostility’, is normally associated with high levels of critical comments. Hence, caregivers who showed high criticism or over-involvement are rated as ‘high EE’ [3–5].

Schizophrenia is one of the most common serious mental disorders that result in changes of perception, emotion, cognition, thinking, and behavior. Both patients and family members often suffer from poor care and social isolation because of widespread ignorance of the disorder. In families with high levels of expressed emotion, the relapse rate for schizophrenia is high [6].

Approximately 50% of patients living with a spouse or their parents had at least one instance of readmission following discharge, compared to only 30% of those living alone [7].

Additional caregiving role to already existing family roles becomes stressful psychologically as well as economically [7]. Unemployment of both patients and families is a major indirect cost, resulting in more than half (61%) of the total economic burden of schizophrenia, these experiences lead family caregivers to have high expressed emotion (HEE), which in turn increases the risk of relapse in persons they are caring for [2].

A prospective study done in Brazil showed that 31% of patients presented relapses and, among the relatives, 68% presented elevated levels of expressed emotion. The proportions of family members with high levels of critical comments and emotional over-involvement were 49% and 52%, respectively [8].

A study conducted in Nigeria showed the prevalence of high expressed emotion to be 50.0%. Relapse rates of people in differing living arrangements after an episode of mental disorder is as follows: 17% for patients living alone or with siblings, 32% for those living with parents and 50% for those living with a spouse [9].

Schizophrenic patients living with relatives having a high expressed emotion (EE) level at hospital admission are more likely to relapse within 9 months after discharge than those patients who are living with relatives showing a low EE level [3].

In a cross-sectional study conducted in Delhi, India schizophrenic patients having families with a high level of critical comments had a threefold greater risk of relapse within 9 months after recovery, and patients with high criticism had a higher chance of early relapse [10].

In the psychiatric in-patient department of the government medical College and hospital Nagpur the total mean score among caregivers of patients with mental illness was 58.12 which indicated high expressed emotions among caregivers, all the demographic factor results the calculated value is less than the tabulated value, so there is no association found between selected demographic variables and EE of the caregivers [11].

In the study conducted at outpatient clinics in Abbasia and Banha Hospitals for Mental Health, it was found that statistically significant relationships existed between patients’ genders and parent EE; it was reported that parents of females made more critical comments than parents of males. More than half of Patients with adolescent-onset had parents rated high criticism [4].

The educational status of the demographic characteristic of patients and relatives was also significantly associated with high EE [5].

A hospital-based cross-sectional study conducted in India among 125 patients revealed that younger patients experienced more EE and patients who were single experienced significantly more EE than married patients, which was similar to the study done in Pakistan [12].

A study done in Nigeria showed that female caregivers were associated with high expressed emotion. It has been found that younger age, female sex, higher educational level, and part-time occupation result in higher levels of psychological distress and distressed caregivers have high expressed emotion [13].

The British studies indicated that, among patients living in “high-EE” homes, the risk of relapse more than doubled for patients who were in face-to-face contact with relatives having high EE 35 h per week or more (69% relapse rate) compared with those (28%) fewer than 35 weekly contact hours [14].

## Methods and materials

### Study area and period

An institutional based cross-sectional study was conducted from April to June 2019 at Jimma University Medical Center (JUMC), which is found in Jimma. Jimma town is found 352 km from southwest of Addis Ababa, the capital city of Ethiopia, providing specialized clinical services to about 15 million people in the catchment. Currently, on average 518 schizophrenic patients are having follow-up at the Psychiatry Outpatient Department (OPD) monthly. At Jimma University Medical Center, a psychiatric clinic was established in 1988 and has been serving more than 10,000 psychiatry patients annually. Currently, the clinic has 26 beds for in-patient service and 04 outpatients department with 2 psychiatrists, 10 psychiatric nurses, 2 clinical psychologists, and 9 M.Sc., 1 Ph.D., and 1 Ph.D. fellow mental health professionals serving about 15 million people in Southwest Ethiopia.

### Participants

Source population: all caregivers of patients with schizophrenia, visiting the Psychiatry Outpatient Department at Jimma University Medical Center.

The sample size was determined using the single population proportion formula by taking the result done in Nigeria; the result of high expressed emotions of caregiver was 50.0%. To get the possible sample at 95% CI that is *Z*–the value of 1.96 and marginal error of 5% is calculated as follows:

*n* = (*Z* *α*/2)^2^ *p* (1−*p*)/*d*^2^, where *n* = number of sample size. *Z* = desired 95% confidence, *Z* = 1.96. *p* = population proportion. *q* = 1− *p* = 1− 0.5 = 0.5, *d* = is the margin of sampling error tolerated (5%).

$$n = \frac{{\left( {1.96} \right)^{2} \left( {0.5} \right)\left( {1 - 0.5} \right)}}{{\left( {0.05} \right)^{2} }},n\;{\text{initial}}\, = \,{384}{\text{.}}$$

By considering 10% (10/100*384 = 38) non-response rate, the final sample size was 422.

Study participants were recruited by consecutive sampling techniques.

Caregivers who were  ≥ 18 years of age and were taking care of patients with schizophrenia were included**.**

### Data collection tool and procedures

A structured questionnaire was developed after reviewing related literature. It was used to collect data about caregivers and patient socio-demographic variables. The last psychiatric diagnosis was taken from medical records of patients. The caregivers Expressed Emotion status was measured by Family Questionnaire (FQ) which is developed by Wiedemann, Rayki, Feinstein, and Hahlweg in 2002, with 20—items which included two domains—Critical Comments CC (10 items—2, 4, 6, 8, 10, 12, 14, 16, 18, 20), and Emotional Over Involvement [EOI] (10 items—1, 3, 5, 7, 9, 11, 13, 15, 17, 19), with maximum value of 40 and the cut-off point for high EE relatives being  > 23 for critical comments and  > 27 for emotional over-involvement [4, 15].

Low EE relatives are those with cutoff point of  ≤ 23 for critical comments and  ≤ 27 for emotional over-involvement. Possible responses of participants is never or very rarely, rarely, frequently, and very frequently, with values ranging from one to four for each response, respectively. The FQ had better agreement with the gold standard questioners of CFI (Camberwell Family Interview) on CC and EOI than did other short EE questionnaires, because both have sensitivity 80%, specificity 70%. Criticism (*α* = 0.86, *n* = 257) and emotional over-involvement (*α* = 0.80, *n* = 256) subscales showed strong internal consistency (22,9, (18) 33,19,34).

### Study variables

The dependent variable of the study was Expressed Emotion (EE) of caregivers (Yes/No).

Independent variables included *caregivers socio-demographic factors* (age, gender, ethnicity, occupation, marital status, educational status, family size, relationship with the patient), distance from hospital in km, and average household monthly income,

Socio-demographic factors of patients: age, gender, marital status, educational status, employment status, the impact of illness on occupation.

Clinical variables: duration of illness, duration of taking care of the patient, time spent with the patient per day, number of the episode, number of previous hospitalizations, number of a family member with psychiatric illnesses, and co-morbid disorder of the patients.

### Operational definitions

High expressed emotion: high EE relatives are with cutoff point of  > 23 for critical comments and  > 27 for emotional over-involvement (22,9,33,19,34).

Low expressed emotion: low EE relatives are with cutoff point of  ≤ 23 of critical comments and  ≤ 27 for emotional over-involvement (22,9,33,19,34).

### Data processing, analysis, and interpretation

The data were checked for consistency and completeness throughout the time of data collection. Data coded and entered twice into double EPI-DATA version 4.41 and then exported to SPSS version 25 for analysis. Before performing binary regression the scores were checked for assumption and whether the model fits or not via Hosmer–Lemeshow. Bivariate regression was computed for each independent variables separately from the dependent variables. Finally, those variables having a *p *< 0.25 were taken to multiple logistic regressions model once, and those with a *p *< 0.05 were considered as having statistically significant association with the dependent variable.

### Ethical consideration

Ethical clearance was obtained from the Institutional Review Board of JU after approval of the proposal. Official permission was collected from Jimma University Medical Center’s psychiatry clinic. The purpose of the study was communicated with study participants and data were collected after written consent is obtained. Caregivers with HEE were consulted with mental health professionals and psychologists working in the unit.

## Results

### Socio-demographic characteristics of study participants

A total of 422 caregivers of patients with schizophrenia have participated in this study. Among study participants, 281 (66.6%) were males, 263 (62.3%) were married, the majority, 313 (74.2%) of respondents were Oromo by ethnicity and 313 (74.2%) were Muslims by religion. The mean age of participants was 40.24 years (SD ± 15.3) and 166 (39.3%) were parents. Nearly one-third (30.6%) of respondents attended primary education. Regarding the occupation of the respondents, 146 (34.6%) were farmers. More than half of the respondents, 228 (54%) live in urban areas, 110 (26.1%) live in a distance of 9–23 km from the Hospital and the median income was 1000 ETB (see Table 1).

**Table 1:** Socio-demographic characteristics of caregiver of patient with schizophrenia at Jimma University Medical Center Psychiatry Clinic, Southwest Ethiopia 2019 (*n* = 422)

Variable	Category	Frequency (*n*)	Percent
Age	18–27	117	27.7
	28–38	97	23.0
	39–52	105	24.9
	52–79	103	24.4
Sex	Male	281	66.6
	Female	141	33.4
Marital status	Single	111	26.3
	Divorced	19	4.5
	Married	263	62.3
	Widowed	29	6.9
Religion	Muslim	313	74.2
	Orthodox	76	18.00
	Protestant	33	7.8
Ethnicity	Amhara	48	11.4
	Oromo	313	74.2
	Tigre yem, gurage, kefa	30	7.1
	Siltea and Dawuro	31	7.3
Educational status	Not able to write and read	87	20.6
	Only able to write and read	28	6.6
	Primary education	129	30.6
	Secondary education	91	21.6
	Higher education and above	87	20.6
Occupation	Farmer	146	34.6
	House wife	43	10.2
	Merchant	56	13.7
	Gov’t employee	57	13.5
	Private employee	42	10.00
	Student	33	7.8
	Retired and unemployed	30	7.1
	Others*	13	3.1
Average household monthly Income in ETB	< 200	114	27.0
	201–1000	162	38.4
	1001–2000	58	13.7
	> 2000	88	20.9
Place of residence	Rural	194	46
	Urban	228	54
Relation to the patient	Parents	166	39.3
	Child	44	10.4
	Siblings	151	35.8
	Aunt/uncle	22	5.2
	Spouse	24	5.7
	Others**	15	3.6

^*^Others (occupation)—daily laborer

^**^Others (relation)—half brothers/sisters, neighborhood, grandchildren

### Caregivers perceived difficulties during caregiving

Of the total respondents, 164 (38.9%) were more than seven family sizes. Three hundred seventy-one (87.9%) had only one family member with mental illness. The mean duration of caregiving was 5.7 (SD ± 4.18) years and the mean length of stay with the patient per 24 h was 7.49 (SD ± 6.24) h. More than half of respondents, 260 (61.8%) had reported no objective burden and 173 (41%) had reported severe subjective burden. Nearly all respondents had reported low perceived stigma 410 (97.2%). Out of the total respondents, 185 (43.8%) had low social support. Nearly all of the participants 97.9% (*n* = 413) reported not having a mental illness and 86.7% (*n* = 366) did not have chronic medical/physical illness which is reported by participants as diagnosed by a health professional (see Table 2).

**Table 2:** Perceived difficulties during care giving and health status among caregiver of patient with schizophrenia at Jimma University Medical Center Psychiatry Clinic, Southwest Ethiopia 2019 (*n* = 422)

Variable	Frequency (*n*)	Percent
Family size		
≤4 family	130	30.8
5–6 family	128	30.3
> 7 family	164	38.9
Family size with MI		
1 family with MI	371	87.9
> 2 family with MI	51	12.1
Duration of take care of patient		
< 2 years	121	28.7
3–5 years	118	28.0
6–8 years	82	19.4
> 8 years	101	23.9
Relative’s hours per day spent in contact with the patient		
< 3 h	129	30.6
4–6 h	126	29.9
7–12 h	117	27.7
> 12 h	50	11.8
Distance from hospital in km		
< 8 km	106	25.1
9–23 km	110	26.1
24–50 km	107	25.4
> 50 km	99	23.5
Report of medical/physical illness		
Yes	55	13.0
No	367	87.0
Report of mental disorder		
Yes	8	1.9
No	414	98.1

### Socio-demographic characteristics of the patients

The median age of the patient was 30 years and nearly one-third, 131 (31%) of the patient’s age was 25 and below. More than half, 310 (73.5%) were males. Most of the patients 271 (64.2%) were single and almost one-fourth, 109 (25.8%) were married. One hundred eighty (42.7%) of patients attended primary education and 157 (37.2%) were unemployed. About 176 (41.7%) patients had stopped their jobs due to the illness (see Table 3).

**Table 3:** Socio-demographic characteristics of patient with schizophrenia at Jimma University Medical Center Psychiatry Clinic Southwest Ethiopia 2019 (*n* = 422)

Variable	Frequency (*n*)	Percent
Age		
13–25 years	131	31.0
26–30 years	102	24.2
31–40 years	96	22.7
40–96 years	93	22.0
Sex		
Male	310	73.5
Female	112	26.5
Marital status		
Single	271	64.2
Divorced	30	7.1
Married	109	25.8
Widowed	12	2.8
Educational status		
Unable to read and wright, only read and wright	89	21.1
Primary education	180	42.7
Secondary education	108	25.6
Higher education and above	45	10.7
Occupation		
Farmer	101	23.9
Housewife	47	11.1
Merchant	18	4.3
Gov’t and private employee	44	10.4
Student	38	9.0
Unemployed	157	37.2
Others*	17	4
Impact of the illness on occupational status		
Unemployed due to illness	50	11.8
Working full time	77	18.2
Working part time	114	27.0
Retired and stop working	181	42.9

^*^Others, daily laborers

#### Clinical characteristics of the patient

Out of the total patients, 70 (16.6%) had a co-morbid neuropsychiatric and medical disorder in summation. Of this, 33 (7.8%) had substance use disorder as reviewed from their medical record. The mean duration of illness was 6.13 (SD ± 5.18) years and the mean age of the first onset of illness was 26.28 (SD ± 13.2) years. On the other hand, 295 (69.9%) had 1–2 episodes and 259 (61.4) of patients had no history of admission (see Table 4).

**Table 4:** Clinical characteristics of patient with schizophrenia at Jimma University Medical Center Psychiatry Clinic, Southwest Ethiopia 2019 (*n* = 422)

Variable	Frequency (*n*)	Percent
First onset of illness		
≤ 18 years	110	26.1
19–23 years	115	27.3
24–30 years	97	23.0
> 30	100	23.7
Number of episode		
1–2 episodes	295	69.9
3–4 episodes	58	13.7
> 4episodes	69	16.4
Hospital admission		
Yes	163	38.6
No	259	61.4
Number of admission		
None	259	61.4
1 admission	102	24.2
2 admission	25	5.9
3 admission	19	4.5
4 admission	17	4
Duration of illness		
< 2 years	145	34.4
3–5 years	79	18.7
6–10 years	133	31.5
> 10 years	65	15.4
Patient’s co-morbid diagnosis		
Yes	70	16.6
No	352	83.4

### Status of expressed emotions among caregivers of patients with schizophrenia

Of the total study participants, 101 (23.9%) reported high critical comments (CC) and 148 (35.1%) reported high emotional over-involvement (EOI). Overall, the status of expressed emotion among caregivers as measured by considering either high CC or high EOI, 184 [43.6% (38.5–48.6)] had higher expressed emotion (see Table 5).

**Table 5:** The Family Questionnaire (FQ) sub-scale among caregiver of patient with schizophrenia at Jimma University Medical Center Psychiatry Clinic, Southwest Ethiopia 2019 (*n* = 422)

Family Questionnaire (FQ) components for assessment of expressed emotion	Frequency (*n*)	Percent
Critical comments		
Low critical comment	321	76.1
High critical comment	101	23.9
	422	100.0
Emotional over-involvement		
Low emotional over-involvement	274	64.9
High emotional over-involvement	148	35.1
	422	100.0
Expressed emotion status		
Low expressed emotion	238	56.4
High expressed emotion	184	43.6
Total	422	100.0

### Factors associated with expressed emotions among caregivers of patients with schizophrenia

#### Bivariate analysis of factors associated with expressed emotion

Those who gave care for about 6–8 years were found to be 2.4 [COR = 2.373, 95% CI (1.335, 4.218)] Participants who were from a household with monthly income  > 2000 ETB were nearly 2 [COR = 1.711, 95% CI (0.976, 2.999)]. Patients who had 3–4 episode were 2.3 times [COR = 2.382, 95% CI (1.339, 4.236)] (see in Table 6).

**Table 6:** Bivariate analysis of factor associated and Status of expressed emotion among caregiver of patient with schizophrenia at Jimma University Medical Center Psychiatry Clinic, Southwest Ethiopia 2019 (*n* = 422)

Variable	Category	Frequency (%)	Expressed emotion status		*P* value	COR (95% CI)
			High EE (*n* = 184)	Low EE (*n* = 238)		
Care giver age	18–27	117 (27.7)	52 (28.3%)	65 (27.3%)	0.323	1.313 (0.765, 2.253)
	28–38	97 (23.0)	44 (23.9%)	53 (22.3%)	0.283	1.362 (0.775, 2.395)
	39–52	105 (24.9)	49 (26.6%)	56 (23.5%)	0.200*	1.436 (0.826, 2.496)
	52–79	103 (24.4)	39 (21.2%)	64 (26.9%)	1	
Caregiver occupation	Farmer	146 (34.6)	54 (29.3%)	92 (38.7%)	1	
	House wife	43 (10.2)	21 (11.4%)	22 (9.2%)	0.165*	1.626 (0.819, 3.229)
	Merchant	56 (13.7)	27 (14.7%)	31 (13.0%)	0.209*	1.484 (0.802, 2.747)
	Gov’t employee	57 (13.5)	28 (15.2%)	29 (12.2%)	0.115*	1.645 (0.886, 3.053)
	Private employee	42 (10.00)	20 (10.9%)	22 (9.2%)	0.216*	1.549 (0.775, 3.096)
	Student	33 (7.8)	13 (7.1%)	20 (8.4%)	0.796	1.107 (0.510, 2.403)
	Retired and unemployed	30 (7.1)	16 (8.7%)	14 (5.9%)	0.099*	1.947 (0.882, 4.299)
	Others	13 (3.1)	5 (2.7%)	8 (3.4%)	0.916	1.065 (0.332, 3.420)
Average monthly income in ETB	< 200	114 (27.0)	47 (25.5%)	67 (28.2%)	1	
	201–1000	162 (38.4)	69 (37.5%)	93 (39.1%)	0.821	1.058 (0.651, 1.719)
	1001–2000	58 (13.7)	20 (10.9%)	38 (16.0%)	0.392	.750 (0.389, 1.448)
	> 2000	88 (20.9)	48 (26.1%)	40 (16.8%)	0.061*	1.711 (0.976, 2.999)
Place of residence	Rural	194 (46)	78 (42.4%)	116 (48.7%)		
	Urban	228 (54)	106 (57.6%)	122 (51.3%)	0.195*	1.292 (0.877, 1.904)
Relation to the patient	Parents	166 (39.3)	68 (37.0%)	98 (41.2%)	1	
	Child	44 (10.4)	15 (8.2%)	29 (12.2%)	0.408	0.745 (0.372, 1.495)
	Siblings	151 (35.8)	76 (41.3%)	75 (31.5%)	0.095*	1.460 (0.936, 2.277)
	Aunt/uncle	22 (5.2)	8 (4.3%)	14 (5.9%)	0.680	0.824 (0.328, 2.071)
	Spouse	24 (5.7)	9 (4.9%)	15 (6.3%)	0.747	0.865 (0.358, 2.090)
	Others	15 (3.6)	8 (4.3%)	7 (2.9%)	0.356	1.647 (0.570, 4.756)
Family size	< 4	130 (30.8)	62 (33.7%)	68 (28.6%)	0.442	
	5–6	128 (30.3)	56 (30.4%)	72 (30.3%)	0.525	0.853 (0.522, 1.393.)
	> 7	164 (38.9)	66 (35.9%)	98 (41.2%)	0.201*	0.739 (0.464, 1.175)
Family size with mental illness	1	371 (87.9)	168 (91.3%)	203 (85.3%)	1	
	> 2	51 (12.1)	16 (8.7%)	35 (14.7%)	0.063*	0.552 (0.295, 1.033)
Duration of take care of pt	< 2 years	121 (28.7)	41 (22.3%)	80 (33.6%)	1	
	3–5 years	118 (28.0)	51 (27.7%)	51 (28.2%)	0.139*	1.485 (0.880, 2.508)
	6–8 years	82 (19.4)	45 (24.5%)	45 (15.5%)	0.003*	2.373 (1.335, 4.218)
	> 8 years	101 (23.9)	47 (25.5%)	47 (22.7%)	0.056*	1.698 (.987, 2.922)
Caregiver chronic physical/medical illness	Yes	55 (13.0)	19 (10.3%)	36 (15.1%)	1	
	No	367 (87.0)	165 (89.7%)	202 (84.9%)	0.149*	1.548 (.856,2.799)
Patients age	13–25 years	131 (31.0)	54 (29.3%)	77 (32.4%)	0.706	1.110 (.645, 1.912)
	26–30 years	102 (24.2)	47 (25.5%)	55 (23.1%)	0.299	1.353 (.765, 2.394)
	31–40 years	96 (22.7)	479 (25.5%)	49 (20.6%)	0.157*	1.519 (.852, 2.707)
	40–96 years	93 (22.0)	36 (19.6%)	57 (23.9%)	1	
Patients occupation	Farmer	101 (23.9)	36 (19.6%)	65 (27.3%)	1	
	House wife	47 (11.1)	21 (11.4%)	26 (10.9%)	0.294	1.458 (0.721, 2.950)
	Merchant	18 (4.3)	8 (4.3%)	10 (4.2%)	0.478	1.444 (0.523, 3.986)
	Gov’t and private employee	44 (10.4)	27 (14.7%)	17 (7.1%)	0.005*	2.868 (1.381, 5.955)
	Student	38 (9.0)	16 (8.7%)	22 (9.2%)	0.483	1.313 (0.613, 2.813)
	Unemployed	157 (37.2)	70 (38.0%)	87 (36.6%)	0.155*	1.453 (0.868, 2.430)
	Others	17 (4)	6 (3.3%)	11 (4.6%)	0.978	0.985 (0.336, 2.885)
Co morbid diagnosis of patients	Yes	70 (16.6)	36 (19.6%)	34 (14.3%)	1	
	No	352 (83.4)	148 (80.4%)	204 (85.7%)	0.150*	0.685 (0.410, 1.146)
Number of episode	1–2	295 (69.9)	115 (62.5%)	180 (75.6%)	1	
	3–4	58 (13.7)	35 (19.0%)	23 (9.7%)	0.003*	2.382 (1.339, 4.236)
	> 4	69 (16.4)	34 (18.5%)	35 (14.7%)	0.119*	1.520 (0.898, 2.575)
Number of admission	None	259 (61.4)	117 (63.6%)	142 (59.7%)	1	
	1 admission	102 (24.2)	34 (18.5%)	68 (28.6%)	0.041*	0.607 (0.376, .980)
	2 admission	25 (5.9)	13 (7.1%)	12 (5.0%)	0.514	1.315 (0.578, 2.991)
	3 admission	19 (4.5)	12 (6.5%)	7 (2.9%)	0.136*	2.081 (0.794, 5.45)
	4 admission	17 (4)	8 (4.3%)	9 (3.8%)	0.880	1.079 (0.404, 2.884)
Duration of illness	≤ 2 years	145 (34.4)	52 (28.3%)	93 (39.1%)	1	
	3–5 years	79 (18.7)	34 (18.5%)	45 (18.9%)	0.292	1.351 (0.772, 2.366)
	6–10 years	133 (31.5)	66 (35.9%)	67 (28.2%)	0.021*	1.762 (1.090, 2.848)
	> 10 years	65 (15.4)	32 (17.4%)	33 (13.9%)	0.069*	1.734 (0.958, 3.138)

NB ^*^P < 0.25

#### Independent predictors of expressed emotions among caregivers of patients with schizophrenia at JUMC

Duration of giving care for about 6–8 years was [AOR = 2.439, 95% CI (1.308, 4.549)] caregivers report of no diagnosis of chronic medical/physical illness were [AOR = 2.274, 95% CI (1.174, 4.406)] and patients who had 3–4 episode were 2.3times [AOR = 2.281, 95% CI (1.253, 4.150)] were demonstrated to have a statistically significant association with caregivers high expressed emotion.

The odds of having high expressed emotion among those who gave care for the patient for about 6–8 years were 2.4 times higher than those who gave care for about  < 2 years.

The odds of having high expressed emotion were 2.2 times higher in those who had no chronic medical/physical illness than those who had chronic medical/physical illness.

Finally, the odds of having high expressed emotion among those caregivers who had patients with 3–4 episodes of illness were 2.3 times higher than those who had 1–2episode of illness (see Table 7).

**Table 7:** Multivariable logistic regression analysis of factors associated with high expressed emotions among caregivers of patient with schizophrenia at Jimma University Medical Center Psychiatry Clinic, Southwest Ethiopia 2019 (*n* = 422)

Variables	Category	Frequency (%)	Expressed emotion status		Multivariable result AOR (95% CI)	*P*-value
			High expressed emotion (*n* = 184)	Low expressed emotion (*n* = 238)		
Duration of taking care of the pt	< 2 years	121 (28.7)	41 (22.3%)	80 (33.6%)		1
	3–5 years	118 (28.0)	51 (27.7%)	51 (28.2%)	1.529 (0.890,2.626)	0.124
	6–8 years	82 (19.4)	45 (24.5%)	45 (15.5%)	2.313 (1.261,4.242)	0.007*
	> 8 years	101 (23.9)	47 (25.5%)	47 (22.7%)	1.467 (0.797,2.701)	0.218
Caregivers report of chronic medical/physical illness	Yes	55 (13.0)	19 (10.3%)	36 (15.1%)		1
	No	367 (87.0)	165 (89.7%)	202 (84.9%)	2.135 (1.143,3.987)	0.017*
Number of episode	1–2	295 (69.9)	115 (62.5%)	180 (75.6%)		1
	3–4	58 (13.7)	35 (19.0%)	23 (9.7%)	0.007*	2.281 (1.253, 4.150)
	> 4	69 (16.4)	34 (18.5%)	35 (14.7%)	0.183	1.504 (0.825, 2.741)

1 = reference value

*NB* ^*^P < 0.05 statistically significant

## Discussion

A total of 422 caregivers of patients with schizophrenia were included in this study. The proportion of high expressed emotion (EE) was 43.6% which is consistent with similar studies conducted in Nigeria (41.4%) [16]. The discrepancy may be due to use of a different assessment tools and sample size.

Regarding the duration of patient care, those who give care for about 6–8 years 82 (19.4%) were 2.4 times more likely to have high expressed emotion than those who give care for about  ≤ 2 years. A study done in Cairo showed that caregivers do not practice any activities and hobbies; this might be due to the caregivers allocation of their lots of time with the patient to provide care and hence the majority of caregivers were spending more than 12 care hours per day and this leads them to have HEE [4]. The possible explanation for this might be that patients with schizophrenia may not be able to carry out their daily activities on their own and hence depends more on their caregivers. Consequently, family caregivers are likely to evaluate their life as being filled with interruptions.

This belief of the caregivers about their own inability to manage severe symptoms might make them encounter repetitious long-term stress, causing them to have the reactions or behaviors found in the HEE. Similarly, the study in northern India showed that caregivers who had sustained distress were likely to show high EE and also had a longer care giving history [3].

A participant who had history of medical/physical illness diagnosis by health professionals was 2.2 times more likely to have high expressed emotion than who had not. This might be due to the fact caregivers with medical/physical illness being responsible for having follow-up program for the patients, and helping with the day to day activities of the schizophrenic patient since these patients have difficulties in self-helping behavior. In relation to this caregivers might get exhausted and show HEE than caregivers who does not have a history of medical/physical illness.

Another explanation might be because those caregivers who have medical/physical illness might not take responsibility to take care of the schizophrenic patient or spend more time with them since the caregivers themselves have their own illness. This leads them to have a short time contacting the patient, therefore they become less likely to have HEE.

Those caregivers of patients who had 3–4 episodes of illness were 2.3 times more likely to have high expressed emotion than those who had 1–2 episode of illness. Consistently with the current study a meta-analysis identified 27 articles reporting EE and psychiatric relapses in schizophrenia patients and confirmed that EE is a good predictor of schizophrenia relapses, especially in patients in the most chronic phase of the disease, current study result found no significant association between relapse and HEE [17].

## Conclusions

In our study, nearly half of caregivers (43.6%) had high expressed emotion. Having 3–4 episodes of illness, Duration of giving care for 6–8 years, caregiver’s report of no diagnosis of medical illness had demonstrated a statistically significant association with caregivers expressed emotion. This study highlights the presence of high expressed motion among caregivers of patients with schizophrenia in our setup. Such findings can be used as a guideline for screening vulnerable family caregivers who have more influential factors of expressed emotion, Therefore, the Ethiopia policy direction can address not only the patient’s mental health, but also the caregiver-expressed emotion.

Mental health professionals are supposed to assess expressed emotion among caregivers of a schizophrenic patient. Therefore, they can conduct psychotherapy to promote the capability of family caregivers to reappraise their situations and experiences, so that they can more effectively manage the stress of caregiving situations of their family members with schizophrenia.

It also helps the caregivers in lowering their expressed emotions and enhancement in their coping strategy. The findings of the present study can be eventually utilized to bring a reduction in a negative atmosphere in caregivers where there is a patient with schizophrenia, like expressed emotion.

As the research design was cross-sectional, the interpretation of causal relationships must be done with caution and preferably, a longitudinal study should be undertaken to verify the credibility of the study findings. Qualitative study since expressed emotion culturally influenced, patient perception of expressed emotion and components of expressed emotion, is going to be considered for next study.

## Data Availability

The data elements utilized in this research work are accessible from the corresponding authors on a rational request.

## Abbreviations

CC: Critical comments
CFI: Camberwell Family Interview
DALYs: Disability-adjusted life years
EE: Expressed emotion
EOI: Emotional over-involvement
FBIS: Family Burden Interview Schedule
FQ: Family Questionnaire
H: Hostility
HEE: High expressed emotion
LEE: Low expressed emotion
MMAS: Morisky Medication Adherence Scale
PDD: Perceived devaluation and discrimination
YLD: Years lived with disability
